# A Machine Learning Model for Predicting Major Depressive Disorder Using Diffusion-Tensor Imaging Data

**DOI:** 10.1192/j.eurpsy.2024.1283

**Published:** 2024-08-27

**Authors:** J. H. Lee, D.-K. Lee

**Affiliations:** Department of Mental Health Research, National Center for Mental Health, Seoul, Korea, Republic Of

## Abstract

**Introduction:**

Major Depressive Disorder (MDD) stands as a prevalent psychiatric condition within the general population. Despite extensive research efforts, the identification of definitive diagnostic biomarkers for depressive disorders remains elusive. Currently, machine learning methods are gaining prominence in the diagnosis of medical illnesses.

**Objectives:**

This study aims to construct a machine learning-based prediction model for Major Depressive Disorder (MDD) by harnessing diffusion tensor imaging (DTI) data.

**Methods:**

The DTI datasets comprising MDD (N=83) and Healthy Control (N=70) groups were procured from the cohort study of Anxiety and Depression conducted at the National Center for Mental Health in South Korea. A machine learning method using a decision tree algorithm was employed to select relevant brain regions and establish a robust diagnostic model. Features associated with white matter (WM) tracts were chosen through recursive feature elimination.

**Results:**

Demographic characteristics, including age, sex, and handedness, displayed no significant differences between the MDD and Healthy Control groups. However, the total score of the Beck Depression Inventory was notably higher in individuals with MDD compared to Healthy Controls. A diagnostic model was crafted using the decision tree algorithms to distinguish between the two groups. The model demonstrated the following classification performance metrics: accuracy (65.6% ± 8.5), sensitivity (66.6% ± 12.5), and specificity (64.7% ± 13.6). Furthermore, through recursive feature elimination, specific neuroanatomical features tied to brain structures such as the inferior cerebellar peduncle, posterior thalamic radiation, cingulum (hippocampus), uncinate fasciculus, and tapetum were identified.

**Conclusions:**

Despite of limited performance of classification, a machine learning-based approach could provide insights into the development of a diagnostic model for MDD using neuroimaging data. Furthermore, these features, derived from DTI-derived data, may have implications for understanding the neural underpinnings of major depressive disorder.

**Disclosure of Interest:**

None Declared

